# Investigation of the Frequency of the Development of Chronic Pain After Thoracotomy

**DOI:** 10.3390/jcm15052035

**Published:** 2026-03-06

**Authors:** Ferda Yaman, Dilek Çetinkaya, İlker Uğurlu, Erhan Durceylan

**Affiliations:** 1 Department of Anesthesiology and Reanimation, Faculty of Medicine, University of Eskişehir Osmangazi, Eskişehir 26040, Turkey; dceyhan@ogu.edu.tr; 2 Anesthesiology and Reanimation Clinic, Ministry of Health Mustafakemalpaşa Hospital, Bursa 16550, Turkey; iugurlu@ogu.edu.tr; 3 Department of Thoracic Surgery, Faculty of Medicine, University of Eskişehir Osmangazi, Eskişehir 26040, Turkey; edurceylan@ogu.edu.tr

**Keywords:** thoracotomy, chronic post-thoracotomy pain, postoperative pain, pain management, self-medication, quality of life, regional anesthesia

## Abstract

**Background**: Chronic pain following thoracotomy remains a common and clinically significant complication that adversely affects functional recovery and quality of life. Despite advances in perioperative analgesic techniques, chronic post-thoracotomy pain continues to be under-recognized and insufficiently managed in routine clinical practice. In this study, we aimed to determine the incidence of chronic pain after thoracotomy and evaluate its impact on daily activities and postoperative pain management behaviors. **Methods**: This retrospective observational study was conducted after institutional ethics committee approval was received (approval no. 2023/61). Patients aged ≥15 years who underwent thoracotomy between 15 June 2022 and 15 June 2023 and had undergone an operation at least three months prior to the study were included. Patients who underwent video-assisted thoracoscopic surgery were excluded. Demographic, surgical, anesthetic, and postoperative analgesia data were obtained from medical records. Patients were contacted by telephone to assess pain intensity using a Numeric Rating Scale (NRS), functional impact on daily activities, and analgesic medication use. The primary outcome was the incidence of chronic post-thoracotomy pain, defined as pain persisting beyond three months and reported at the time of the interview. **Results**: A total of 56 patients were included in the analysis. Chronic pain was reported by 55.4% of the patients. Pain that interfered with daily activities and required medication use was reported by 51.5% of the patients. Thirty-three patients (57.9%) reported an NRS score > 3 during movement. Among patients with chronic pain, 64.7% reported self-medication without physician consultation, whereas only 11.8% sought medical advice for pain management. **Conclusions**: Chronic pain remains highly prevalent after thoracotomy and substantially interferes with daily functioning. A considerable proportion of patients self-manage their pain without medical supervision, underscoring the need for structured postoperative follow-up, early identification of high-risk patients, and individualized multimodal analgesic strategies to reduce the burden of chronic post-thoracotomy pain.

## 1. Introduction

Thoracotomy remains a fundamental surgical approach for accessing the thoracic cavity and continues to be widely performed, particularly in complex oncologic and advanced thoracic procedures. Among various techniques, posterolateral thoracotomy is considered one of the most painful surgical incisions due to extensive muscle division and rib retraction involving major thoracic musculature [1]. Despite advances in surgical techniques and perioperative analgesic strategies, postoperative pain following thoracotomy remains a significant clinical challenge.

Although minimally invasive approaches such as video-assisted thoracoscopic surgery (VATS) and robotic-assisted thoracic surgery have been associated with reduced acute postoperative pain and lower rates of chronic postsurgical pain compared with open thoracotomy, chronic post-thoracotomy pain syndrome (CPTPS) continues to be reported in contemporary cohorts [2,3]. Recent studies demonstrate that persistent pain remains prevalent even in the era of enhanced recovery protocols and advanced regional anesthesia techniques, with substantial inter-study variability in reported incidence rates [4,5].

The present study focuses exclusively on patients undergoing open thoracotomy, a procedure that remains clinically relevant in many institutions worldwide. Contemporary data evaluating predictors of chronic postsurgical pain in open thoracotomy cohorts remain limited, particularly with respect to patient-reported outcomes, healthcare-seeking behavior, and real-world pain management patterns [6,7]. Although regional anesthesia techniques such as thoracic epidural analgesia and fascial plane blocks have been shown to improve acute postoperative pain control after thoracotomy, their impact on the development of chronic postsurgical pain remains controversial. Effective management of acute pain is considered a potential modifiable factor in preventing central sensitization and chronic pain development. However, despite advances in perioperative analgesia, chronic post-thoracotomy pain continues to be reported at clinically relevant rates. This discrepancy underscores the importance of evaluating the real-world incidence and functional consequences of chronic pain following thoracotomy.

According to the International Association for the Study of Pain (IASP), chronic pain is defined as pain persisting for more than three months [2]. Thoracotomy has consistently been associated with one of the highest incidences of chronic postsurgical pain among major surgical procedures [8]. Recent single-center and regional analyses further confirm that chronic pain remains a substantial postoperative burden following open thoracotomy [9].

The development of chronic post-thoracotomy pain is multifactorial and has been associated with demographic variables, perioperative factors, psychological vulnerability, and analgesic strategies [5,10]. Although thoracic epidural analgesia, paravertebral blocks, erector spinae plane blocks, and multimodal analgesic protocols are widely employed, evidence regarding their long-term effectiveness in preventing chronic pain remains heterogeneous [7]. Importantly, persistent postoperative pain significantly restricts daily activities and negatively impacts long-term quality of life [8].

In routine clinical practice, regional anesthesia techniques are frequently recommended to mitigate acute postoperative pain and potentially reduce the risk of chronic pain development. However, the real-world incidence of chronic post-thoracotomy pain, its impact on daily functioning, and patients’ pain management behaviors—including self-medication and patterns of physician consultation—remain insufficiently characterized.

The primary aim of this retrospective study was to determine the incidence of chronic post-thoracotomy pain in patients at least three months after surgery.

Secondary aims were to evaluate its impact on daily activities and functional recovery, and to assess analgesic consumption patterns among affected patients.

## 2. Materials and Methods

### 2.1. Study Design and Ethical Approval

This retrospective observational study was conducted in accordance with the principles of the Declaration of Helsinki (1975, revised in 2013) and approved by the Eskisehir Osmangazi University Faculty of Medicine Ethics Committee (Approval Code: 2023/61; Approval Date: 20 June 2023). Verbal informed consent was obtained from all participants prior to the telephone interview. This retrospective observational study was designed to investigate the incidence and risk factors associated with chronic pain following thoracic surgery. Demographic characteristics, perioperative variables, and postoperative pain outcomes were systematically collected and analyzed. Surgical approach, including open thoracotomy and minimally invasive techniques, was also evaluated as a potential determinant of chronic pain development. A summary of the study design and methodological framework is presented in Table 1.

### 2.2. Patient Selection

The hospital database was reviewed to identify patients aged ≥15 years who had undergone thoracotomy between 15 June 2022 and 15 June 2023. Patients were included if, at the time of the assessment, at least three months had elapsed since surgery. Exclusion criteria were age < 15 years, incomplete medical records, and surgery performed using a video-assisted thoracoscopic approach. The choice between open thoracotomy and VATS was determined by the operating surgeon based on tumor characteristics (size, location, and local invasion), disease stage, and technical feasibility. Open thoracotomy was generally performed in cases requiring extended resection, central tumor localization, or suspected vascular involvement, whereas VATS was preferred for peripheral and technically suitable lesions. Due to the retrospective and exploratory nature of this study, a formal a priori power calculation was not performed. The study included a convenience sample of all eligible patients (n = 56) treated at our center within the specified timeframe to provide a preliminary evaluation of chronic pain prevalence.

### 2.3. Anesthesia and Perioperative Management

Standard institutional anesthesia protocols were applied to all patients. General anesthesia was induced with propofol and maintained using a balanced technique consisting of O_2_/air/sevoflurane and remifentanil infusion. Intraoperative anesthesia was standardized across all patients, and remifentanil infusion was administered according to institutional protocol in all cases. A neuromuscular blockade was achieved with rocuronium at a dose of 0.5 mg/kg.

Intraoperative analgesia was induced via remifentanil infusion. In patients in whom preoperative epidural analgesia had been induced, intraoperative epidural administration was not routinely continued because of the risk of hypotension.

Before extubation, all patients were administered 1 g of paracetamol intravenously and 100 mg of tramadol.

Epidural analgesia, when used, was employed preoperatively. Fascial plane blocks (erector spinae plane block or serratus anterior plane block) were employed at the end of surgery according to institutional practices.

### 2.4. Postoperative Pain Management

The choice of postoperative analgesic technique (thoracic epidural versus regional block) was determined by the attending anesthesiologist based on institutional practice, patient comorbidities, and contraindications to epidural placement. Thoracic epidural analgesia was generally preferred in patients without contraindications, whereas regional techniques were used in cases where epidural placement was unsuitable or declined. For postoperative analgesia, patients who were subjected to a regional block were treated with tramadol infusion (with a maximum daily dose not exceeding 400 mg) and intravenous paracetamol at 6 h intervals.

In patients who underwent a fascial plane block at the end of surgery, intravenous paracetamol was administered at 6 h intervals, and a tramadol bolus of 1 mg/kg was administered if the Numeric Rating Scale (NRS) score exceeded 4.

### 2.5. Surgical Procedure and Postoperative Care

All the patients underwent standard posterolateral thoracotomy, with or without rib resection. Chest tubes were removed when there were no air leaks and pleural drainage was <100 mL over a 24 h period.

### 2.6. Data Collection

Demographic data, comorbidities, previous surgical history (e.g., upper-abdominal or breast surgery), and preoperative medication use, including chronic analgesic and opioid therapy, were recorded from medical records and evaluated as a potential risk factor for chronic postsurgical pain. Intraoperative anesthesia techniques, postoperative pain management strategies, and perioperative complications were obtained from electronic medical records.

### 2.7. Telephone Follow-Up and Outcome Assessment

Patients were contacted chronologically by telephone to obtain additional information regarding postoperative pain and its impact on daily activities. Chronic pain assessment was performed using a structured telephone interview conducted by trained investigators at least three months after surgery. Chronic postsurgical pain was defined according to the International Association for the Study of Pain (IASP) criteria as pain persisting for more than three months after the surgical procedure.

During the interview, patients were systematically asked about the presence of pain localized to the surgical incision or surrounding area, differentiation from preoperative pain, and the exclusion of alternative causes. Pain intensity was quantified using the Numeric Rating Scale (NRS, 0–10), a validated and widely used tool for clinical pain assessment. Patients were also asked whether pain intensity changed with coughing or movement and whether pain affected daily activities or prompted healthcare-seeking behavior. Chronic pain assessment was performed using a structured questionnaire administered via telephone interview by trained investigators to ensure standardized data collection.

During the structured telephone interview, the following standardized questions were asked:

1. Did you experience any pain around the surgical incision or surrounding area three months after surgery?
2. Did you have pain before surgery? If so, was the pain you felt three months after surgery similar to your preoperative pain?
3. Was there any other identifiable cause of the pain?
4. Did you take any measures to relieve pain, such as rest, reducing daily activities, self-medication, or seeking medical assistance?
5. On a scale from 0 (no pain) to 10 (unbearable pain) (Numeric Rating Scale, NRS), how would you rate your pain? Did the intensity change with coughing or movement?

Chronic post-thoracotomy pain was defined as pain around the surgical incision persisting for more than three months after surgery and reported at the time of the telephone interview. Chronic post-thoracotomy pain was defined as pain localized to the surgical area, persisting for ≥3 months after surgery, and not attributable to other identifiable causes. The NRS score reflected pain intensity at the time of the interview.

The primary outcome was the incidence of chronic post-thoracotomy pain as assessed during a follow-up, whereas secondary outcomes included pain intensity, functional impact on daily activities, and postoperative pain management behaviors.

### 2.8. Statistical Analysis

Descriptive statistics were used to summarize the data. Continuous variables were expressed as means ± standard deviations or medians (minimum–maximum), as appropriate. Categorical variables were presented as frequencies and percentages. All statistical analyses were performed using SPSS version 27.0 software (IBM Corp., Armonk, NY, USA).

## 3. Results

**Figure 1:**
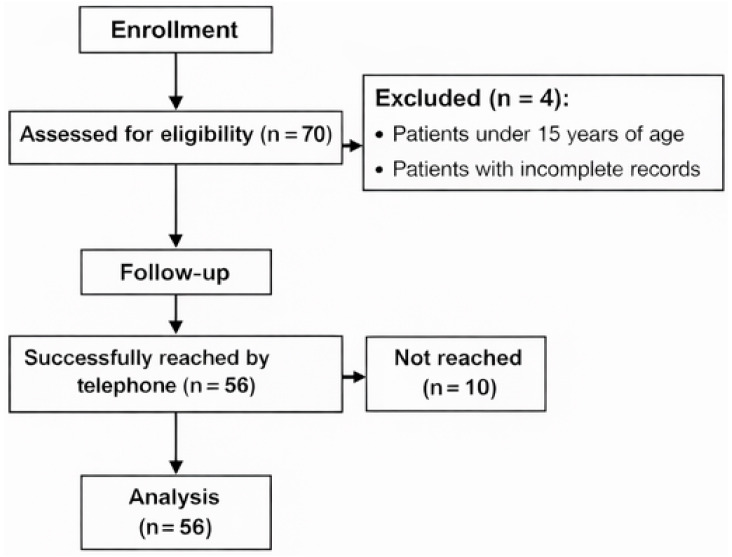
Flow diagram representing study selection.

During the one-year study period, 70 patients who underwent thoracotomy were assessed for eligibility. Four patients were excluded because they were <15 years old or had incomplete medical records. Consequently, 66 patients were eligible for the follow-up. Of these, 10 could not be reached by telephone. Therefore, 56 patients (17 females and 39 males) were successfully contacted and included in the final analysis (Figure 1).

The patients’ demographic and preoperative characteristics are summarized in Table 2.

Surgical indications, previous surgeries potentially affecting chronic pain (e.g., upper-abdominal or breast surgery), the duration of surgery, and thoracotomy-related variables are presented in Table 3.

Postoperative pain management techniques, analgesic medications used, and responses to the chronic pain survey are shown in Table 4.

Chronic pain persisting for more than three months after surgery was reported by 55.4% of the patients. Pain that interfered with daily activities and required medication use was reported by 51.5% of the patients. Thirty-three of the fifty-six patients (57.9%) reported a Numeric Rating Scale (NRS) score > 3 during movement. Among patients with chronic pain, 64.7% reported using analgesic medication independently without physician consultation, whereas only 11.8% sought medical advice for pain management.

## 4. Discussion

The primary objective of this study was to determine the frequency of chronic pain in patients undergoing thoracotomy and to evaluate its impact on daily activities and pain management behaviors. The prevalence of chronic post-thoracotomy pain has been reported to vary widely across studies, reflecting differences in surgical techniques, analgesic strategies, patient populations, and methodological approaches [11,12]. In the present study, chronic pain persisting for more than three months after surgery was observed in 55.4% of patients. This finding is consistent with previous reports and confirms that chronic pain remains a common and clinically relevant complication following thoracotomy.

In the era of minimally invasive thoracic surgery, the burden of chronic post-thoracotomy pain has declined but has not been eliminated. Consistent with the contemporary literature, open thoracotomy remains associated with a higher risk of chronic pain compared with minimally invasive approaches such as VATS [13,14]. The term “nerve-sparing” primarily refers to careful identification and preservation of the intercostal neurovascular bundle during rib retraction and closure. Nevertheless, studies suggest that surgical technique alone does not fully explain the persistence of chronic pain [15].

Although regional analgesic techniques improve acute postoperative pain control, their long-term influence on chronic pain prevention remains heterogeneous across studies [16]. The variability in reported chronic pain outcomes likely reflects heterogeneity in perioperative pain protocols, surgical techniques, and patient-related factors. These observations underscore the need for real-world analyses, such as the present study, to better identify clinically relevant predictors and refine risk stratification strategies.

More than half of the patients in our cohort (51.5%) reported that their pain interfered with daily activities and required medication use. Bayman et al. demonstrated that pain-related limitations in daily activities are common among patients with persistent pain after thoracic surgery [17]. Similarly, prospective data indicate that chronic pain may persist for months to years after thoracic procedures, contributing to long-term functional burden [18]. Together, these findings emphasize that chronic post-thoracotomy pain not only persists over time but also substantially affects quality of life and functional capacity.

An important observation in our study was that 64.7% of patients reported self-medicating for pain relief, whereas only 11.8% sought medical consultation. This low rate of physician consultation may be influenced by pain severity, socio-cultural factors, health literacy, and accessibility of healthcare services. In Türkiye, non-prescription analgesics such as paracetamol and non-steroidal anti-inflammatory drugs are readily available, which may contribute to high rates of self-medication. Globally, self-medication and over-the-counter analgesic use remain widespread, and unsupervised use should not be underestimated [19]. These findings highlight a gap in structured postoperative follow-up and suggest the need for improved patient education regarding appropriate pain management after thoracic surgery.

Chronic post-thoracotomy pain is widely recognized as a multifactorial condition [3]. Studies examining the influence of surgical technique on chronic pain development have produced inconsistent findings. Jiwnani et al. reported that a modified nerve-sparing thoracotomy did not significantly reduce chronic pain compared with standard posterolateral thoracotomy at six months postoperatively [15]. Michel-Cherqui et al. found that although acute pain was less frequent after an axillary approach, the incidence of chronic pain was comparable to that observed after the posterolateral approach [7]. Likewise, prospective analyses have shown similar frequencies and severities of pain following thoracotomy and thoracoscopic procedures at mid-term follow-up [11]. While intercostal nerve injury has traditionally been regarded as a major contributor to chronic post-thoracotomy pain, these findings suggest that additional perioperative and patient-related factors play a substantial role in its development. The choice of anesthetic and analgesic technique appears to be an important determinant in the development of chronic postsurgical pain. While general anesthesia effectively abolishes intraoperative awareness, perioperative factors such as the type of anesthetic technique, intraoperative opioid exposure, and adequacy of acute postoperative pain control may influence central sensitization and long-term pain outcomes. Contemporary clinical studies have demonstrated that perioperative opioid administration and the intensity of early postoperative pain are associated with an increased risk of persistent postsurgical pain development [20,21]. Both inhalational anesthetics and propofol exert analgesic effects by modulating central sensitization pathways; however, inhalational agents alone appear to be insufficient for reducing the prevalence of chronic pain [17]. Propofol additionally interacts with glycine and γ-aminobutyric acid type A receptors and possesses antioxidative properties that may contribute to its analgesic profile [17]. Moreover, high doses of intraoperative remifentanil have been associated with chronic postsurgical pain one year after video-assisted thoracoscopic surgery, suggesting that excessive opioid exposure should be avoided when possible [21]. Although remifentanil-induced hyperalgesia has been described in the literature, particularly with high-dose administration, all patients in our cohort received remifentanil as part of a standardized anesthetic protocol. Therefore, intraoperative opioid exposure was consistent across the study population, limiting its potential role as a confounding factor.

Preemptive analgesia may mitigate central sensitization by blocking the transmission of nociceptive impulses. Preoperative epidural analgesia has been shown to reduce the occurrence and severity of persistent pain syndromes after thoracotomy [22]. Beyond pharmacological interventions, psychological factors such as anxiety, depression, stress responses, and poor sleep quality have been identified as independent predictors of persistent postsurgical pain [23]. Cognitive behavioral therapy for high-risk patients and perioperative patient education regarding expected postoperative discomfort may help reduce fear-avoidant behaviors and improve functional recovery [22].

Genetic predisposition may further modulate the risk of chronic pain in susceptible individuals. Polymorphisms involving catechol-O-methyltransferase (COMT), voltage-gated sodium and calcium channels, and μ-opioid receptors have been implicated as potential genetic contributors to chronic pain [23]. A validated presurgical risk model incorporating six easily measurable factors—type of surgical procedure, younger age, physical and mental health status, preoperative pain at the surgical site, and preoperative pain at another location—has been shown to effectively identify patients at risk of chronic postsurgical pain [24]. Such models could facilitate early, targeted interventions and personalized perioperative pain management strategies.

Future studies should incorporate validated pain and functional interference instruments, such as the Brief Pain Inventory or neuropathic pain questionnaires (e.g., DN4 or PainDETECT), to better characterize pain phenotypes and functional outcomes.

From a clinical perspective, our findings emphasize the need for structured postoperative follow-ups, early identification of high-risk patients, and individualized multimodal analgesic strategies to reduce the long-term burden of chronic post-thoracotomy pain.

From a clinical standpoint, recognizing the risk factors associated with chronic postsurgical pain after thoracic procedures enables early identification of high-risk patients and facilitates implementation of targeted multimodal analgesic strategies. This approach may not only improve acute postoperative pain control but also reduce the long-term burden of chronic pain, aligning with emerging evidence that tailored perioperative protocols contribute to better outcomes [25,26,27].

This study has several limitations. First, its retrospective design precluded the formation of comparison groups and limited causal inference regarding the effects of different surgical or analgesic techniques. Second, intraoperative remifentanil doses varied among patients and could not be standardized. Third, the sample size was relatively small and restricted to a one-year period. Fourth, neuropathic pain was not specifically assessed, and information regarding patients’ socio-cultural characteristics and preoperative anxiety levels was unavailable. Additionally, postoperative follow-up durations varied among patients when pain was assessed. Pain outcomes were based on telephone interviews and self-reported data, which are subject to recall bias and reporting inaccuracies. A limitation of this study is the use of telephone-based follow-up rather than in-person clinical assessment, which may introduce recall bias. However, structured interviews were conducted to minimize variability in responses. Telephone-based assessment is commonly employed in long-term postoperative pain studies where routine outpatient follow-up may not be feasible. The lack of in-person clinical examination further limited the objective assessment of pain characteristics. Although pain intensity was assessed using a validated numeric rating scale and a structured interview format, objective quantitative sensory testing was not performed. Therefore, neuropathic components of chronic pain could not be formally characterized. Additionally, the non-responses from 10 eligible patients may have introduced selection bias, as patients with more severe or milder symptoms may have been more or less likely to participate in the telephone follow-up. The selection of analgesic technique was not randomized but based on clinical judgment and institutional practice, which may introduce selection bias and influence pain outcomes. The relatively long interval between surgery and telephone follow-up (median 14 months) may have introduced recall bias. Patients were not prospectively instructed to record pain intensity, which may limit the precision of retrospective reporting. However, chronic pain was defined as persistent pain present at the time of the interview and persisting for ≥3 months after surgery, rather than relying exclusively on precise recall of pain intensity at a specific early postoperative time point. Nevertheless, this limitation should be considered when interpreting the findings.

## 5. Conclusions

Chronic pain remains a frequent and clinically significant complication following thoracotomy, substantially impairing daily functioning and quality of life. In this study, more than half of the patients experienced persistent pain beyond three months after surgery, and a considerable proportion relied on self-medication without medical supervision. These findings underscore the need for structured postoperative follow-ups, early identification of high-risk patients, and individualized multimodal analgesic strategies to reduce the long-term burden of chronic post-thoracotomy pain. The findings suggest that chronic post-thoracotomy pain remains common in real-world practice and may significantly affect daily functioning, highlighting the need for improved postoperative follow-up and patient education. In conclusion, chronic post-thoracotomy pain appears to remain common in real-world clinical practice and may substantially affect daily functioning in a considerable proportion of patients. A notable number of patients reported self-medication and limited medical consultation.

Given the retrospective design and sample size limitations, these findings should be interpreted as descriptive observations. Further prospective studies are warranted to better characterize risk factors and long-term functional outcomes.

Given the multifactorial nature of post-thoracotomy pain syndrome, preventive efforts should begin in the preoperative period and incorporate individualized risk assessment, appropriate regional anesthesia techniques, and perioperative psychological support. Despite existing interventions aimed at alleviating chronic pain, its prevalence remains unacceptably high. Future prospective studies and personalized pain management approaches are warranted to reduce the burden of chronic post-thoracotomy pain and improve long-term patient-centered outcomes.

## Figures and Tables

**Table 1:** Summary of Study Design and Methodology.

Component	Description
**Study design**	Retrospective observational study
**Setting**	Single-center tertiary hospital
**Study period**	15 June 2022–15 June 2023
**Population**	Patients ≥ 15 years undergoing open thoracotomy (n = 56)
**Exclusion criteria**	Age < 15, incomplete records, VATS
**Chronic pain definition**	Pain ≥ 3 months (IASP criteria)
**Assessment method**	Structured telephone interview
**Pain intensity tool**	Numeric Rating Scale (0–10)
**Primary outcome**	Incidence of chronic post-thoracotomy pain
**Secondary outcomes**	Pain severity, functional impact, healthcare-seeking behavior

**Table 2:** Demographic and preoperative characteristics of the patients.

		Min–Max	Median	Mean ± SD/n-%	
Age		15.0–78.0	57.0	57.2 ± 14.2	
Gender	Female			17	25.8%
	Male			49	74.2%
BMI		15.1–40.4	24.8	25.1 ± 5.1	
ASA	I			6	9.1%
	II			32	48.5%
	III			28	42.4%
Comorbidities	(−)			19	28.8%
	(+)			47	71.2%
HT				21	31.8%
COPD				16	24.2%
CAD				15	22.7%
Oncological disease				14	21.2%
DM				12	18.2%
Rheumatological disease				5	7.6%
Thyroid treatment				4	6.1%
Renal disease				2	3.0%
Cerebral disease				1	1.5%
Medication use	Absent			54	81.8%
	Present			11	16.7%
	Unknown			65	98.5%
Antidepressant				6	54.5%
Steroid				6	54.5%
NSAID				2	18.2%
Paracetamol				1	9.1%
Opioid				1	9.1%

Data are presented as means ± standard deviations or n (%). Abbreviations: SD, standard deviation; BMI, body mass index; ASA, American Society of Anesthesiologists; COPD, chronic obstructive pulmonary disease; CAD, coronary artery disease; NSAID, non-steroidal anti-inflammatory drug.

**Table 3:** Surgical data of the patients.

		Min–Max	Median	Mean ± SD/n-%	
Surgical history	Absent			53	80.3%
	Present			12	18.2%
	Unknown (patient file could not be reached)			1	1.5%
Upper abdominal				7	58.3%
Thoracotomy				5	41.7%
Breast surgery				1	8.3%
Indications for thoracotomy	Lobectomy			39	59.1%
	Decortication			6	9.1%
	Pleural biopsy			5	7.6%
	Wedge resection			5	7.6%
	Cyst excision			2	3.0%
	Cystectomy			2	3.0%
	Nodule excision			2	3.0%
	Pneumonectomy			2	3.0%
	Biopsy			1	1.5%
	Chest wall resection			1	1.5%
	Hydatid cyst			1	1.5%
	Rib resection			1	1.5%
	Segmentectomy			1	1.5%
	Liquid drainage			1	1.5%
Thoracic side	Right			42	63.6%
	Left			23	34.8%
	Bilateral			1	1.5%
Duration of surgery (min)		150.0–420.0	270.0	249.8 ± 81.6	

Data are presented as means ± standard deviations or n (%). Abbreviations: SD, standard deviation.

**Table 4:** Characteristics of chronic post-thoracotomy pain.

		Min–Max	Median	Mean ± SD/n-%	
Postoperative month		7.0–19.0	14.0	13.5 ± 3.4	
Postoperative block	No block			35	55.6%
	Epidural			8	12.7%
	ESP			13	20.6%
	SAPB			1	1.6%
	PVB			5	7.9%
	Intercostal Block			2	3.2%
Postoperative pain management	No			5	7.6%
	NSAID			55	83.3%
	Tramadol infusion			44	66.7%
	Paracetamol			55	83.3%
	Paracetamol + NSAID			1	1.5%
Did you experience any pain around the surgical incision or surrounding area three months after surgery?	No			17	25.8%
	Yes			36	54.5%
	Unknown			13	19.7%
Did you have pain before surgery? Was the pain three months after surgery the same as the preoperative pain?	No			43	65.2%
	Yes			10	15.2%
	Unknown			13	19.7%
Same				1	10.0%
Less than				2	20.0%
More than				7	70.0%
Was there any other cause for the pain?	No			48	72.7%
	Yes			5	7.6%
	Unknown			13	19.7%
Did you take any measures to relieve pain, such as rest, reducing daily activities, self-medicating, or seeking medical assistance?	No			19	28.8%
	Yes			34	51.5%
	Unknown			13	19.7%
Self-medication				22	64.7%
	Doctor’s prescription			4	11.8%

Data are presented as means ± standard deviations or n (%).Percentages were calculated based on the number of patients reporting any preoperative pain (n = 10). Percentages were calculated based on the number of patients who took any measures to relieve pain (n = 34). Abbreviations: SD, standard deviation; ESP, erector spinae plane block; SAPB, serratus anterior plane block; PVB, paravertebral block; NSAID, non-steroidal anti-inflammatory drug.

## Data Availability

The data presented in this study are available from the corresponding author upon reasonable request.

## Abbreviations

The following abbreviations are used in this manuscript:

ASA	American Society of Anesthesiologists
BMI	body mass index
SD	standard deviation
ESP	erector spinae plane block
IASP	International Association for the Study of Pain
SAPB	serratus anterior plane block
PVB	paravertebral block
NSAID	non-steroidal anti-inflammatory drug
HT	hypertension
COPD	chronic obstructive pulmonary disease
CAD	coronary artery disease
DM	diabetes mellitus
